# Interleukin-6 Producing Pheochromocytoma: A Rare Cause of Systemic Inflammatory Response Syndrome

**DOI:** 10.1155/2019/7906272

**Published:** 2019-03-24

**Authors:** Nelson Carvalho Cunha, Leonor Gomes, Joana Saraiva, Isabel Paiva

**Affiliations:** Department of Endocrinology, Hospitais da Universidade de Coimbra, Centro Hospitalar e Universitário de Coimbra, 3075 Coimbra, Portugal

## Abstract

Systemic inflammatory response syndrome (SIRS) can be a rare manifestation of pheochromocytoma, since this adrenal tumor may produce cytokines and other hormones or neuropeptides besides catecholamines. We report the case of a 53-year-old female patient with a pheochromocytoma that presented with fever and weight loss of 5% in one month along with normocytic anemia, thrombocytosis, leukocytosis, and elevated C-reactive protein. In this setting, interleukin-6 (IL-6) was requested and was elevated [26.7ng/L (<7.0)]. She also presented biochemical evidence of ACTH-independent cortisol production without overt Cushing syndrome. After adrenalectomy, the inflammatory syndrome resolved and all biochemical parameters normalized, including IL-6 and ACTH. To our knowledge, this is the first case report of IL6-producing pheochromocytoma along with autonomous cortisol production.

## 1. Introduction

Pheochromocytoma is a tumor that arises from chromaffin cells of adrenal medulla and usually presents with paroxysms of hypertension, palpitations, sweating, and headache due to excessive catecholamine release. However, some of these tumors may secrete other hormones or neuropeptides, resulting in unusual clinical manifestations and hindering the diagnosis. Some of these are cytokines, mainly interleukin-1 (IL-1), IL-6, tumor necrosis factor-*α* (TNF-*α*), and interferon-*γ* (INF- *γ*), resulting in systemic inflammation [1]. Fever of unknown origin is uncommon in patients with pheochromocytoma, but there are few reports of patients with systemic inflammatory response syndrome (SIRS) and elevated IL-6 plasma levels due to this adrenal tumor [1–9]. The SIRS consists in a pathophysiologic response to a nonspecific insult and is characterized by fever or hypothermia, tachycardia, tachypnea, and/or leukocytosis [10]. IL-6 is a cytokine that regulates immune responses and plays a major role in inflammatory cascade by stimulating the release of acute-phase reactants such as C-reactive protein (CRP) [11]. IL-6 also regulates hematopoiesis and stimulates hypothalamic-pituitary-adrenal axis [12]. It is produced mainly by lymphocytes and monocytes and is physiologically expressed in the adrenal cortex, but not in adrenal medulla [13]. In this report, we describe a patient with a pheochromocytoma and elevated plasma IL-6 that presented with SIRS.

## 2. Case Presentation

A 53-year-old woman was referenced from primary care physician to her local hospital due to fever at evening and a weight loss of 5% in one month. The patient had a previous history of total hysterectomy and atrophic gastritis and was under proton pump inhibitors. No relevant familial history was known.

At admission, she was pale, with 37.9°C of temperature, normal blood pressure (119/69mmHg) and heart rate (91/min), and 61 Kg of weight (BMI 25.5Kg/m^2^), without Cushing syndrome signs or other relevant clinical findings.

Patient's laboratory tests at admission (Table 1) revealed marked thrombocytosis (platelets 743x10^9^/L), normocytic anemia (Hb 10.1 g/dL), and slight leukocytosis (WBC 10.2x10^9^/L) with elevated ferritin, C-reactive protein levels [(22.74 mg/dL (<0.5)], and normal procalcitonin. No pathogenic agents were found on blood, urine, and cerebrospinal fluid cultures nor alcohol-acid resistant bacilli. Viral serological markers were also negative. She also presented elevated fasting blood glucose (130mg/dL) and A1C of 6.4% as well as slight elevation of liver enzymes and decreased albumin. Brain tomography showed no alterations and echocardiogram showed slightly enlarged left atrium and normal left ventricular ejection fraction.

**Figure 1:**
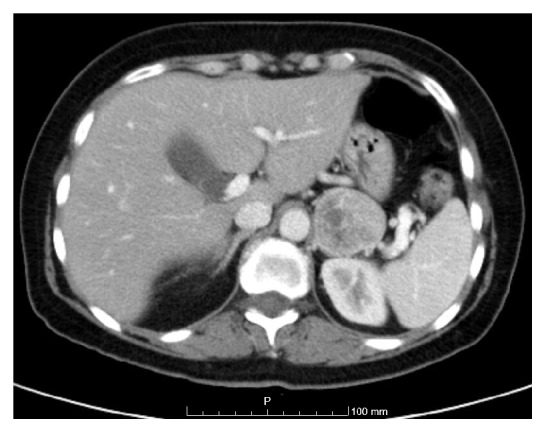
*Adrenal tumor on abdominal CT scan*. Adrenal tumor with 4.2cm length and heterogeneous iodine contrast uptake.

The patient was initially medicated with levofloxacin during 7 days but without any improvement. Then, a thoracic and abdominal tomography (CT) was performed and revealed a nodular lesion of 4.2 cm length, with heterogeneous contrast enhancement, not clear if the origin was the gastric fundus or the left adrenal (Figure 1). Upper digestive endoscopy showed a hiatus hernia and erosive antral gastritis, without any suspicious features. The abdominal magnetic resonance imaging revealed a left adrenal tumor with 4.3cm length, hyperintense in T2-weighted images and hypointense in T1, with peripheral contrast enhancement and increased washout, possibly indicating a pheochromocytoma. The patient was then referred to our department and when asked, she also referred 3 to 4 episodes per day of palpitations, sweating, headache, and limbs paresthesia, mainly related with efforts, which were not initially considered. At biochemical evaluation, she presented elevation of plasma and urinary normetanephrine [3503 pg/mL (<120) and 5505 ug/24h (50-650) resp.] with plasma and urinary metanephrine and 3-methoxythyramine within reference range (Table 1). Adrenal androgens were also normal. However, ACTH was suppressed [<5pg/mL (9-52)] with asleep plasma midnight cortisol [7.1ug/dL (<1.8)], urinary free cortisol [96ug/24h (10-80)], and cortisol after 1 mg overnight dexamethasone suppression test (3.7ug/dL) slightly elevated, consistent with autonomous cortisol production. ^125^I-metaiodobenzyl guanidine (MIBG) scintigraphy showed an accumulation of the isotope in the left adrenal tumor. After integration of these clinical, laboratory, and imaging data, the diagnosis of pheochromocytoma was made. In the presence of a SIRS in a patient with a newly diagnosed pheochromocytoma, the measurement of plasma IL-6 was requested and it was elevated [26.7 ng/L (<7.0)], consistent with IL-6-producing pheochromocytoma. Biochemical screening of MEN2 syndrome was negative.

Patient started preoperative alpha-blockade treatment with phenoxybenzamine 10 mg id that was increased to 10 mg 2id after one week and beta-blockade treatment was added with propranolol 10 mg id. During treatment, her minimum BP was 86/53mmHg and maximum was 116/57mmHg and minimum heart rate was 73/min and maximum was 115/min. She remained without fever during this period. After 23 days of alpha-blockade single-port laparoscopy, left adrenalectomy was performed without complications. Histological examination showed a well-delimitated pheochromocytoma with 3.0x2.5x1.8cm and potential malignant biological behavior with PASS score = 11 (Table 2) (potential malignant biological behavior if PASS score ≥ 6 [14]). Immunohistochemistry revealed strong positivity to chromogranin A, synaptophysin, and neuron-specific enolase and negativity to cytokeratin AE1/AE3, calretinin, and inhibin A. Ki67 proliferation index was 1-2%. After surgery, the alpha- and beta-blockade was stopped and due to autonomous cortisol production with suppressed ACTH, hydrocortisone was initially prescribed at stress dose and then tapered to 20 mg per day.

In reevaluation two months after surgery she had no complaints. Plasma and urinary normetanephrine were within reference range and IL-6 was undetectable (Table 1). Total blood count and inflammatory parameters have all normalized. Hydrocortisone was tapered to suspension just before revaluation and a short Synacthen® test was performed, which excluded adrenal insufficiency (plasma cortisol 60 minutes after 250 *μ*g of tetracosactide: 19 mg/dL). Abdominal CT and MIBG scintigraphy had no evidence of persistent disease. A genetic test was requested and no germline mutations were detected in the following genes: RET, VHL, SDHAF2, SDHB, SDHC, SDHD, MAX, and TMEM127.

## 3. Discussion

This case shows a very rare presentation of an inflammatory syndrome of unknown origin in a patient with a newly diagnosed pheochromocytoma and elevated levels of IL-6 which resolved after adrenalectomy.

Although she presented some paroxysmal events of palpitations, sweating, headache, and limbs paresthesia, the main clinical sign was the fever accompanied by the elevation of inflammatory markers, normocytic anemia, and thrombocytosis, which delayed the correct diagnosis. The lack of improvement after antibiotic therapy and the nonidentification of pathogenic agent led to the identification of an adrenal tumor on abdominal tomography and consequently to the diagnosis of a pheochromocytoma. The presence of an inflammatory syndrome in a patient with a newly diagnosed pheochromocytoma led us to measure the plasma IL-6 which was significantly elevated.

This patient also presented suppressed plasma ACTH with slightly elevated midnight plasma cortisol, urinary free cortisol, and plasma cortisol after overnight dexamethasone suppression test consistent with autonomous adrenal cortisol secretion. It is described that IL-6 can stimulate HPA axis by increasing ACTH secretion, either directly or by augmenting the effect of CRH [15], as well as adrenal cells to produce cortisol [16]. However, its mechanism is not fully understood. To the extent of our knowledge, this is the first published pheochromocytoma producing IL-6 with associated autonomous cortisol secretion.

After adrenalectomy, the inflammatory syndrome resolved as well as all blood and biochemical parameters, including normetanephrine, C-reactive protein, IL-6, and ACTH. Hydrocortisone was prescribed at stress dose during adrenalectomy and no complications were noted. The dose was tapered and stopped until the short Synacthen® test was done which excluded adrenal insufficiency.

Another major point in this case is the potential risk of malignancy, since some histological features of malignancy as well as predominant normetanephrine secretion were present. It is known that pheochromocytomas with malignant behavior are associated with a predominant secretion of normetanephrine [17]. However, all of the reported IL-6-producing pheochromocytomas were found to secrete predominantly norepinephrine and none were described as malignant. After one year of follow-up, our patient presented no clinical, biochemical, or imaging evidence of relapse or distant metastases. Germline mutations in genes associated with malignant behavior were absent, lessening the malignancy potential [18].

This case shows a very rare presentation of a pheochromocytoma with associated IL-6 production and accompanied by autonomous cortisol secretion. The clinical presentation of an inflammatory syndrome of unknown origin may hamper the correct diagnosis, being extremely important to be aware of this condition to do the prompt diagnosis and the potentially curative treatment.

## Figures and Tables

**Table 1:** Laboratory data before and after left adrenalectomy.

	At diagnosis	After surgery	Reference range
*Interleukin-6 (ng/L)*	*26.7*	*<1.5*	*<7.0*

Plasma metanephrine (pg/mL)	35.9	13.1	<60

Urinary metanephrine (ug/24h)	76.5	22.1	30-350

Plasma normetanephrine (pg/mL)	3503.4	92.5	<120

Urinary normetanephrine (ug/24h)	5505.0	105.1	50-650

Plasma 3-methoxythyramine (pg/mL)	10.4	<10.0	<14

Urinary 3-methoxythyramine (ug/24h)	749.8	87.6	30-300

ACTH (pg/mL)	<5.0	19	9-52

Plasma cortisol 8am (ug/dL)	15	8.5	(5-25)

Asleep plasma cortisol 12pm (ug/dL)	7.1	1.0	<1.8

Urinary free cortisol (ug/24h)	96		10-80

Hemoglobin (g/dL)	10.1	13.8	12.0-15.0

MCV (fL)	84.3	86.5	83.0-101.0

WBC (x10^9^/L)	10.2	6.0	4.0-10.0

Platelets (x10^9^/L)	743	190	150-400

C-reactive protein (mg/dL)	22.74	0.11	<0.5

Procalcitonin (ng/mL)	0.11		<0.5

Ferritin (ng/mL)	566	65	15-150

Fasting blood glucose (mg/dL)	130	88	60-109

A1C (%)	6.4	5.1	4.0-6.0

Albumin (g/dL)	3.3	3.6	3.5-5.2

Calcitonin (pg/mL)	<2.0		<10

PTH (pg/mL)	68		9-72

25-hydroxyvitamin D (ng/mL)	18		>29

Calcium (mg/dL)	8.8	9.4	8.8-10.6

ACTH, adrenocorticotropic hormone; MCV, mean corpuscular volume; WBC, white blood cells; GGT, gamma-glutamyl transferase; PTH, parathyroid hormone.

**Table 2:** Pheochromocytoma of the Adrenal Gland Scaled Score by Thompson.

Feature	Points assigned	Presence in patient's tumor
Large nests or diffuse growth (>10% of tumor volume)	2	Yes

Central (middle of large nests) or confluent tumor necrosis	2	Yes

High cellularity	2	Yes

Cellular monotony	2	No

Tumor cell spindling	2	No

Mitotic figures > 3/10HPF	2	No

Atypical mitotic figures	2	No

Extension into adipose tissue	2	Yes

Vascular invasion	1	No

Capsular invasion	1	Yes

Profound nuclear pleomorphism	1	Yes

Nuclear hyperchromasia	1	Yes

*Total*	*20*	*11*

HPF: high-power field.

## References

[1] Minetto M., Dovio A., Ventura M. (2003). Interleukin-6 producing pheochromocytoma presenting with acute inflammatory syndrome. *Journal of Endocrinological Investigation*.

[2] Salahuddin A., Rohr-Kirchgraber T., Shekar R., West B., Loewenstein J. (1997). Interleukin-6 in the fever and multiorgan crisis of pheochromocytoma. *Scandinavian Journal of Infectious Diseases*.

[3] Fukumoto S., Matsumoto T., Harada S.-I., Fujisaki J., Kawano M., Ogata E. (1991). Pheochromocytoma with pyrexia and marked inflammatory signs: A paraneoplastic syndrome with possible relation to interleukin-6 production. *The Journal of Clinical Endocrinology & Metabolism*.

[4] Suzuki K., Miyashita A., Inoue Y. (1991). Interleukin-6-producing pheochromocytoma. *Acta Haematologica*.

[5] Shimizu C., Kubo M., Takano K. (2001). Interleukin-6 (IL-6) producing phaeochromocytoma: direct IL-6 suppression by non-steroidal anti-inflammatory drugs. *Clinical Endocrinology*.

[6] Yarman S., Soyluk O., Altunoglu E., Tanakol R. (2011). Interleukin-6-producing pheochromocytoma presenting with fever of unknown origin. *Clinics (Sa˜o Paulo, Brazil)*.

[7] Tokuda H., Hosoi T., Hayasaka K., Okamura K., Yoshimi N., Kozawa O. (2009). Overexpression of protein kinase C-delta plays a crucial role in interleukin-6-producing pheochromocytoma presenting with acute inflammatory syndrome: a case report. *Hormone and Metabolic Research*.

[8] Takagi M., Egawa T., Motomura T. (1997). Interleukin-6 secreting phaeochromocytoma associated with clinical markers of inflammation. *Clinical Endocrinology*.

[9] Kang J. M., Lee W. J., Kim W. B. (2005). Systemic inflammatory syndrome and hepatic inflammatory cell infiltration caused by an interleukin-6 producing pheochromocytoma. *Endocrine Journal*.

[10] Balk R. A. (2014). Systemic inflammatory response syndrome (SIRS): where did it come from and is it still relevant today?. *Virulence*.

[11] Tanaka T., Narazaki M., Kishimoto T. (2014). IL-6 in inflammation, immunity, and disease. *Cold Spring Harbor Perspectives in Biology*.

[12] Papanicolaou D. A. (2000). Interleukin-6: the endocrine cytokine. *The Journal of Clinical Endocrinology & Metabolism*.

[13] Päth G., Bornstein S. R., Ehrhart-Bornstein M., Scherbaum W. A. (1997). Interleukin-6 and the interleukin-6 receptor in the human adrenal gland: expression and effects on steroidogenesis^1^. *The Journal of Clinical Endocrinology & Metabolism*.

[14] Strong V. E., Kennedy T., Al-Ahmadie H. (2008). Prognostic indicators of malignancy in adrenal pheochromocytomas: clinical, histopathologic, and cell cycle/apoptosis gene expression analysis. *Surgery*.

[15] Chrousos G. P. (1995). The hypothalamic-pituitary-adrenal axis and immune-mediated inflammation. *The New England Journal of Medicine*.

[16] Ciacciarelli M., Bellini D., Laghi A. (2016). IL-6-producing, noncatecholamines secreting pheochromocytoma presenting as fever of unknown origin. *Case Reports in Medicine*.

[17] Kimura N., Takekoshi K., Naruse M. (2018). Risk stratification on pheochromocytoma and paraganglioma from laboratory and clinical medicine. *Journal of Clinical Medicine*.

[18] Lenders J. W. M., Duh Q.-Y., Eisenhofer G. (2014). Pheochromocytoma and paraganglioma : an endocrine society clinical practice guideline. *The Journal of Clinical Endocrinology & Metabolism*.

